# Meeting Highlights: Plant, Animal and Microbe Genomes X

**DOI:** 10.1002/cfg.168

**Published:** 2002-04

**Authors:** Jo Wixon, Jo Dicks

**Affiliations:** 1 MRC UK HGMP-RC Hinxton, Cambridge UK; 2 John Innes Centre Norwich Research Park Colney Norwich UK


**Comparative and Functional Genomics**
*Comp Funct Genom* 2002; **3**: 178–193.
Published online 14 March 2002 in Wiley InterScience (www.interscience.wiley.com). **DOI:** 10.1002/cfg.168

**Feature**

### Meeting Highlights: Plant, Animal and Microbe Genomes X

12th–17th January 2002, San Diego, USA

Jo Wixon[1] and Jo Dicks[2]
[1] *MRC UK HGMP-RC, Hinxton, Cambridge, UK*
[2] *John Innes Centre, Norwich Research Park, Colney, Norwich, UK*

### Plenary sessions

**Francis Collins (NIH, USA)** started the first plenary session with a talk entitled **Large-scale sequencing: Past, Present and Future**. He recounted the history of the human genome project. The project will achieve all of the goals set out by the NRC panel in 1988, in spring 2003. They will have 99% coverage of the euchromatin, no centromeres, and a small number of gaps, due to uncloneable regions (they are working on new technologies to overcome this problem).

Observations that came from the draft sequence include that we have only 30–40 000 genes, that the male mutation rate is twice that of females and that 'junk DNA' contains remnants of the raw materials of evolution.

He prefers to say that we are in the 'genome era' rather than the 'post-genome era', as we don't know everything about the genome yet. The next phase of HGP will involve medical, comparative and functional genomics, and proteomics. He feels that their main investment is most likely to be in the technologies required for these approaches, as none of the existing ones are good enough to allow scale-up yet.

The mouse genome is being sequenced using a combination of whole genome shotgun and clone-by-clone BAC approach, and is going to be finished to a high level. He thinks it is unlikely that this level of finishing will be done for another mammalian genome. The Trans NIH BAC sequencing effort, which includes a BAC construction project, is a key sign of this change in emphasis. These projects cover all animals, fungi and eukaryote protists. He also highlighted a call by NHGRI for white papers proposing organisms for genome sequencing (other than eubacteria, archaea and plants). He expects that 5–10 years of vigorous activity in large-scale sequencing should cover most important organisms. Then, he said, we will be in the 'post-sequencing era'!

**Paul Keim (Northern Arizona University, USA)** gave a presentation on **Anthrax**, which he described as a very poor pathogen, with a very good toxin. The ability of its spore form to stay viable in the soil for long periods of time prompted its selection as a biological weapon in the USA, UK and Russia. Global collections of bacterial isolates show very low variation, which could be because it is recently evolved, or because it evolves very slowly, which could be caused by the spore stage.

TIGR have almost completed the sequence of the 5.5 Mb chromosome and the two plasmids of *B. anthracis* and are moving onto other isolates. His group have been identifying the regions with the most variation, which are the tandem repeats. They use PCR with flanking primers to fingerprint strains, testing a huge number of loci per colony. Of the 55 VNTR loci they use, the 8 most hypervariable regions are population markers. In 1000 genotyped isolates, they have seen just over 200 unique genotypes. In the phylogenetic tree they see a major type (A) found in North America, the UK and Turkey, the animal vaccine strain clusters with this group. The B group are predominantly from South Africa and there is also a very rare B2 group. In the US they see a correlation of anthrax presence with cattle trails, which they believe may be the original route of spread.

**Michael Bevan (JIC, UK)** spoke about **the *Arabidopsis* genome** and comparisons to other plant genomes. The genome was finished at the end of 2000; about 60% of the predicted genes had similarity to genes of known function and were assigned putative functions. *Arabidopsis* has more

Copyright © 2002 John Wiley & Sons, Ltd.

genes than *Drosophila* and *C. elegans*, but the same types of families. It has more large families and many tandem arrays of genes. Large regions were seen to be duplicated providing evidence for at least one whole genome duplication. He highlighted the work of other groups on comparative genomics. Renate Schmidt's comparisons to *Capsella* show that the two genomes are very similar, with conserved gene order. Ian Bancroft has made comparisons to *Brassica* genomes, which show some conservation, but more differences than in *Capsella*. Todd Vision and Steve Tanksley have made comparisons with tomato finding some highly conserved regions and in one region, a ratio of one tomato region to four *Arabidopsis* regions was found.

He is involved in a project to generate knock-outs of almost all *Arabidopsis* genes. To generate their mutants they are using a combination of T-DNA and transposon insertional mutagenesis; with the average gene being 5 kb, they need 100 000 mutant lines. There are other *Arabidopsis* functional genomics projects, AFGC (USA), Genoplante (France), Garnet (UK), GABI (Germany) and the EU REGIA and EXOTIC projects. He coordinates the EXOTIC project, which is studying gene regulation using a gene trap approach. Tools for integrating the data from all these projects with each other, and the seed collections are under development.

**David Relman (Stanford University, USA)** spoke about **detecting human commensals and pathogens using genomic tools**. In assays of the human subgingival crevice (the gap between tooth and gum) using broad domain rDNA PCRs, over 50% of the sequences found were novel and included some Archaea, and some representatives of bacterial phylotypes that were not thought to suit the environment. Comparing this survey with previous studies indicates that it is unlikely that all the organisms from this habitat have yet been found. Even healthy blood, which has long been thought to be 'bug' free, can show the presence of bacterial rDNA. He suggests that a catalogue of these bacteria could help to spot pathogens against the background. He would also like to see catalogues of more complex flora such as those in the mouth and on the skin. He is interested in variations in flora between individuals, and is working in collaboration with researchers from LLNL, ANL and Washington University on the development of a high density rDNA array. He is also using arrays to look at host gene expression patterns during infection, to look for diagnostic markers of infection. The results show some shared responses but also organism specific responses, and there are also differences between the responses to live and killed bacteria, these are likely to be the responses to toxins. Interestingly, in attempts to establish a 'normal' profile for healthy peripheral blood using their arrays, they found that the anonymous clustering performed on the data separated males and females.

**Stephen Goff (Syngenta)** gave a presentation entitled **Rice as a model cereal**. The Syngenta TMRI team has generated 6X sequence coverage of ~389 Mb of the rice genome, with the aim of enabling expression profiling, proteomics and identification of candidate genes for traits. In addition to their genome sequence and mapping data, they have sequenced over 330 000 SAGE tags, of which ~45 000 are unique. They also have data on more than 90 000 rice cDNAs (95% of which are represented by their gene predictions) and over 70 000 cDNAs from other cereals. They estimate that the rice genome has ~150 Mb of short repeats, and 35–50 000 genes (based on their high and low confidence gene prediction data).

Comparing the rice data to *Arabidopsis* and the other complete genomes, they see an average identity of 30% at the amino acid level. They found ~8400 genes that were only in the rice and *Arabidopsis* genomes (these may be 'plant specific' genes), and ~3500 genes that were in *Arabidopsis* and not rice. 3291 of these were not found in any of the other species, and he suggests that some of them may not be true genes. They see no obvious long range conservation of gene order, but do see 367 pairs of conserved neighbours at 10 kb spacing, which increases to 850 pairs at 30 kb spacing.

Of 696 maize genes, 680 have a rice homologue at e-9 probability and of 238 wheat genes there are 232 with rice homologues at this cut off. Similar results were seen for rye and barley, with lower numbers for oats and sorghum. The rice-maize macrosynteny map can be used to transfer assignments of QTLs and candidate genes between the genomes. They have made a rice gene chip, with 23 700 rice genes (~15 000 of which have been classified); Maize DNA hits 18 377 of the elements on the array, Barley DNA hits 16 377. They have even been able to use the array to confirm the published barley GA hormone pathway.

**Bob Waterston (Washington University)** opened the second plenary session with a presentation on the **sequencing of the mouse genome**. They currently have 3 × coverage of whole genome shotgun (WGS)

Copyright © 2002 John Wiley & Sons, Ltd.

data and 5-6X of BAC data. They have used automated assembly of BAC fingerprinting data to generate ~9000 contigs, over 500 of these have been manually edited and compared to the human genome. BLAST comparisons of BAC end sequences to the human genome have been used to aid scaffold building in the assembly process, joins are only made when fingerprinting data supports the comparative data. They now have 2.5 Gb of data, giving 89% coverage and have stopped generating WGS data. Most of the contigs are ordered and oriented and the average scaffold size is 0.5 Mb.

The data can be viewed using mouse ensembl at **http://www.ensemblmouse.org/**. Comparing their data to the human genome they see that just over 3% is highly conserved, but only half of this can be accounted for. Some of the unknown areas of homology could be human genes missed by the current annotation, rRNA genes, or regulatory regions; these possibilities are under investigation.

He commented on an analysis by the Haussler group, which looked at synonymous and non-synonymous substitutions in 8000 Refseq genes between human and mouse. They saw a wide range of % synonymous changes, and that the % identity of the 3$^{rd}$ base in four-fold degenerate codons was higher than would be expected and variable. The levels of neutral variation at transposable elements and in ancient repeats exceed the levels found at four-fold degenerate sites.

**Ralph Greenspan** gave a presentation **on gene networks and genetic subtleties of behaviour in *Drosophila***. *Drosophila* mutants have made a large contribution to our understanding of circadian rhythm and learning and memory. Since then, it has been shown that behavioural genes are pleiotropic, for example, the period gene has roles in circadian rhythm, and sensitisation and learning. Looking at natural variation, they found a gene with two alleles affecting the tendency of larvae to move around to find a food source (rover vs sitter). Deletion of the gene was lethal, suggesting that it was pleiotropic.

Array comparisons of rover and sitter larvae, showed variation in many genes with roles in metabolism and others of unknown function. They also looked at geotactic behaviour, which is seen as the preference to move up or down in an upright maze. In array comparisons of flies with positive and negative geotactic behaviour they saw differences in some circadian rhythm genes and some chaperones. In particular, a pigment dispersing factor related to circadian rhythm and the *Cry* circadian rhythm gene were less expressed in negatively geotactic flies.

The team then mapped out the interactions of genes involved in the temperature sensitive passing out response. When they changed the genetic background, they saw that the network of interactions changed significantly, leading them to believe that networks can be very interactive and fluid. Looking at housekeeping enzymes, they see an extensive network of interactions between these genes, which makes them think these may not be such good comparators for array studies. His view is that proteins have a repertoire of functions, which can be recruited at certain times, and responses will not always be the same (they are a little loose). He sees this as degeneracy, rather than redundancy, meaning that the system has a wider application, and can reach the same endpoint in a number of ways. He imagines it as a topographical layout, in which changes in one member of the network produce distortions throughout a system.

**Tom Blundell (University of Cambridge, UK)** spoke about his work towards **understanding the structure and function of the proteome**. He discussed sequence-based structural homology recognition tools, such as Threader, PSSM and FUGUE (produced in his lab). Behind FUGUE is the database HOMSTRAD, with 516 structural alignments of homologous families (including 2055 structures) and 2101 singletons. Each amino acid in a structure has a different substitution pattern and from these they build environment specific substitution matrices (e.g: buried vs accessible). They make structure-based alignments and include the substitution scores and gap penalties etc. to produce profiles. FUGUE outperforms homology only tools such as PSI-BLAST in spotting superfamily members and performs well against CLUSTALW, getting better accuracy when the environmental substitution matrices are added in. FUGUE can also see homologies between families that PSI-BLAST doesn't pick up, in one case highlighting a potential catalytic domain.

Rather than taking the high-throughout, structural genomics approach adopted by several consortia, his group are interested in signal transduction proteins, which act as complexes in networks. They have been studying the fibroblast growth factor receptors, which are alternately spliced, existing in a large number of isoforms. The active kinase is found within the cell, and there

Copyright © 2002 John Wiley & Sons, Ltd. 

are forms with extracellular domains; those with shorter extracellular domains are more sensitive, and are activated in situations where those forms with longer extracellular domains are not. Various combinations of the receptors and FGF are seen in the cell, with different components, some are unstable (typically these are 1 : 1 complexes), some are very stable (these commonly have more components, giving more cooperativity).

**David Galbraith (University of Arizona)** spoke about his work **exploring the nucleus for high-throughput characterisation of transcription**. There are many concerns about existing array profiling techniques and they have tackled these in their work on corn and rice arrays made to study drought and salinity stress responses. Using their corn array, they compared manual and automated hybridisation approaches, finding them similarly accurate. They printed multiple ESTs per gene, with multiple replicates of points, and areas of points, to check for variation and found the levels acceptable. The results from their rice array show that the response breaks up into early and intermediate phases. Their data showed that the choice of time points is crucial, as is replication, they saw that results can be unreproducible, however, genes were consistently up-regulated, or down-regulated, or unaffected, so they feel the technique is more qualitative than quantitative. In their work with *Arabidopsis* arrays they looked at cross hybridisation using the P450 genes. Hybridising single genes to the array showed that they do hit several others of the family, at a lower level of hybridisation. This should not affect strong signals, but would interfere with detection of more subtle responses.

The current approaches for profiling expression sample the polyA RNA present in the cytoplasm, which is a product of both transcription and degradation (translation is also occurring), he would like to look at the nucleus. He also wants to obtain cell type specific data from complex plant tissues, which requires identification and purification of cell types without damaging or disturbing them. They have made NLS tagged GUS fusions and also histone-GFP fusions, with cell specific promoters, which they have shown to specifically label nuclei of the chosen cell types. They use careful disruption of plant tissue followed by flow sorting of labelled nuclei from the transgenic cell protoplasts and isolate mRNA from them for profiling by microarray or SAGE. They do need large numbers of nuclei to obtain enough material, typically over $2 \times 10^5$.

The Animals plenary session started with a talk by **Steve Brown (MRC Mammalian Genetics Unit, UK)** on **mutagenesis and genomics in the mouse**. He spoke about the work of the International Mouse Mutation Consortium, which aims to produce one mutation per gene in the mouse. They hope to identify every gene affecting key traits of biomedical interest by phenotype analyses of the mutants. They will produce and distribute an archive of mutant mice and provide informatics support for the project. Using ENU mutagenesis they can expect to get one mutant for any 1 kb locus in any 1000 mice. They archive tail and sperm samples of each mutant, to allow DNA analysis and 'resurrection' of any chosen mutant. They are performing a genome-wide screen for dominant phenotypes, using a hierarchical system of tests, and have so far identified 1167 abnormal phenotypes, around 500 of which have been shown to be true inherited mutations, overall, they see a 2% incidence of dominant mutations. Using sperm from the collection in IVF, they are performing backcrosses to map the underlying loci. So far, $\sim$80 loci have been mapped, $\sim$72 of which appear to be novel loci.

Several groups have performed gene-driven screens of the UK mouse ENU mutant progeny archive. This is an archive of tail and sperm samples from apparently normal male progeny of ENU mutants, used for detection of recessive mutants. They screen $\sim$2500 mice to have a good chance of finding one mutant allele and 5000 to find several mutant alleles of their gene of interest. This approach is crucial for genes where the knockout mutant is lethal, as it can yield a viable mutant/s, which can be used to study the function of the gene.

**Ian Franklin (CSIRO, Australia)** spoke about **finding genes for wool production and quality**. He gave a detailed introduction to wool biology, describing how it is the follicles that determine most of the important qualities for wool, such as surface area, fibre density, diameter and length. The sheep's coat is made up of a woolly undercoat of secondary follicles, covered by an upper layer of long, straight, coarse primary follicles.

The ovine genome has 27 chromosomes; a map has been constructed with 1061 markers, $\sim$200 of which are genes. QTL mapping has been used to identify some wool related loci and they started analysing skin ESTs 5 years ago. They have four libraries (two adult, two foetal) that have been

Copyright © 2002 John Wiley & Sons, Ltd. 

normalised against total RNA. They have sequenced ~3200 clones, finding genes from most common functional categories, and screened them for dinucleotide repeats. They have performed in situ expression analysis of 1089 ESTs, 82% of which gave non-specific patterns. However, they have found several hair bulb, and other follicle region, specific genes. Included in these are 53 strong signal bulb genes, 86 medium signal bulb genes and 37 fibre cortex genes. Unfortunately they have not as yet found any sebaceous gland or dermal papillae specific genes. There are ~30 genes that are known to be follicle related, but these have not been found in their EST resource.

They have used arrays to compare foetal against adult samples, and normal against EGF treated (used to induce shedding of fleece without need for shearing) sheep, in each case finding a few genes showing marked differences in expression. They are making more arrays and starting to profile the expression patterns of both the known and unknown genes that they have identified as candidates, they also plan to look at their promoter regions.

**Eric Green (NHGRI, USA)** gave the final presentation in this session, describing **a comparative study of selected regions in multiple vertebrates**. Starting with human to mouse comparisons, the NHGRI team defined probes for highly conserved areas within five regions of interest from human chromosome 7. These were used to isolate BAC clones representing these regions from 11 vertebrate species (those with an existing BAC resource). 900 clones covering the five regions are being sequenced and ~600 have already been sequenced, at NISC. Information on the status of the project is hosted at: **https://www.nisc.nih.gov/open_page.html?projects/zooseq.html**. The two most complete regions are the CFTR/WNT2 region on chromosome 7q31 and the Williams syndrome region on 7q11 and 7q22.

Looking at three way comparisons of the data obtained, human vs mouse is very similar to human vs rat, but markedly different from mouse vs rat. Mouse and rat are much more closely related, with lots of non-exon matches. Human, zebrafish and *Fugu* however, triangulate much better, indicating that zebrafish and *Fugu* must be very distant from one another.

The level of conservation of intron sequences varies from gene to gene and also across species. The level of conservation of any one gene in mouse and rat is always less than for that gene in cow, pig, cat and dog, but there are genes for which the level of conservation in the rodents is higher than the levels of conservation of some other genes in cow, pig, cat and dog. On a plot of sequence conservation against evolutionary distance from man, mouse and rat cluster together, but are outliers, with lower than expected conservation, which could be due to their much shorter generation time. Using multidimensional scaling analysis with the overall DNA identity in alignments for a target region, they see that, as in the other graph, human, baboon and chimp cluster, as do mouse and rat, but zebrafish and *Fugu* are separated, reflecting the results of the triangulation analysis. The team will evaluate how well shotgun data performs against finished data for these comparisons, to help decide how many genomes will need to be finished. They also plan to widen the project to other vertebrates, filling in the evolutionary gaps between the mammals and chicken, and between chicken and the fish, with marsupials, monotremes and reptiles.

### Comparative genomics

**Lei Liu, (W. M. Keck Center for Comparative and Functional Genomics, USA)** spoke about COMPASS II, a web-based resource for **prediction of gene map location based upon a parallel cattle-human radiation hybrid mapping**. This tool operates on the theory that, having a comparative map between two species, the location of a gene in one species can be predicted if it is conserved and if its location is known in the other species. The first version of this tool predicted the cattle chromosome on which genes were located with 95% accuracy. This version improves upon that by incorporating radiation hybrid (RH) map data to allow for prediction of chromosomal region and by providing a web-based interface. The process involves identifying putative homologues by BLASTN of human UniGene, retrieval of various fields of UniGene annotation and prediction of cattle chromosome and map bin. The tool can be adapted to any pair of species with adequate RH map data.

**John Quackenbush (TIGR, USA)** described **the TIGR Gene Indices**, a set of resources that link ESTs, genes, and genomes. These gene indices (available at: **http://www.tigr.org/tdb/tgi.shtml**) provide assembled consensus sequences (TCs) of high confidence, that represent their best estimate of the genes underlying the ESTs. There are currently indices for almost 50 species, including human,

Copyright © 2002 John Wiley & Sons, Ltd. 

mouse, rat, *Drosophila*, zebrafish, rice, tomato, potato, maize, soybean, *Arabidopsis*, yeasts and a variety of eukaryotic parasites. Comparisons between these resources have been used to generate TOGA, a resource of orthologous clusters with alignments. They are also working on a project to provide comparative maps linking completed genomic sequences to the TCs (see **http://www.tigr.org/tdb/map.shtml**). A further project, RESOURCERER provides annotation based on the gene indices for commonly available microarray resources. It also allows comparisons between resources from the same species using either the TGI or UniGene, and between species using the TOGA database. The group aim to increase the frequency with which the indices (and TOGA) are updated, with a planned shift to three updates a year.

**Clemens Suter-Crazzolara (LION Bioscience, Germany)** provided an overview of tools available from Lion Bioscience for **computational comparative genomics**, in particular ArraySCOUT™, which was used by the *Listeria* sequencing consortium to manage the data produced in the recent comparison of *Listeria monocytogenes* with *Listeria innocua* (see Suter-Crazzolara and Kurapkat, this issue).

**Ramesh Kantety (Cornell University, USA)** described the construction of **a comparative genetic map of cultivated tef** using RFLP anchor probes from barley, maize, millets, rice, oat and wheat. Tef (*Eragrostis tef*) is a major cereal crop in Ethiopia. It has superior grain nutritional quality to most cereals, and is relatively resistant to damage from pests and pathogens, unfortunately though, it has low yield potential due to crop lodging. The genetic comparative map will be the first step towards crop improvement for Tef. The group are constructing EST libraries to supply markers for the map and aim to provide *in silico* prediction of their orthologous map location in other grasses. They are also undertaking a QTL study to identify genes underlying traits such as crop yield and lodging. In addition, candidate genes, such as those known to be involved in lignin biosynthesis and dwarfing are being analysed on the Tef mapping population to find their association with yield and lodging.

**Wusirika Ramakrishna (Purdue University, USA)** described how **comparative sequence analysis of two regions in grass genomes** has identified frequent genetic rearrangements. The team has sequenced BAC clones covering the *Rp1* disease resistance locus and the *Waxy1* (*Wx1*) locus in a range of grasses (see Ramakrishna *et al.* this issue).

**Shanna Moore (Boyce Thomson Institute for Plant Research, USA)** described the development and utilization of tomato microarrays for the solanaceae. This group have produced a tomato microarray with 9200 elements that will be available to the public in the near future. They have used the array to study fruit ripening and development and have shown that the array can be used for other *Solanacae*, such as peppers and potatoes (see Moore *et al.*, this issue).

### Functional genomics

**Satoshi Tabata (Kazusa DNA Research Center, Japan)** spoke about their analyses of *Lotus japonicus*. They used a SAGE approach to study the root to nodule transition, identifying stage specific sequences, several of which have been verified by RT-PCR. The sequences are compared to their EST resources to identify genes, where no match is obtained, they use 3′ RACE to obtain longer fragments. They now plan to apply this approach to other tissues. They have generated an EST library from seed points and are sequencing 3′UTRs of their clones. In addition they are using genes from other legumes to PCR screen genomic libraries, clones identified are sequenced for gene prediction. They have TAC and BAC libraries of the genome and are end sequencing 640 clones. Based on the 914 genes identified so far, the average *L. japonicus* gene is 2.7 kb long, 76% of genes have introns, the average intron length being 380 bp and the average exon being 276 bp long. Other work includes searching clones for SSRs and using PCR testing for polymorphism in the parents of the mapping population. They are also looking for SNPs. For more information about the project, visit: **http://www.kazusa.or.jp/lotus/**.

**Khalid Meksem (Southern Illinois** University, USA) spoke about his work in **integrating physical maps and genetic markers in a range of plants**. His group are developing BAC libraries and physical maps for plants including soybean, *Arabidopsis*, *Lotus* and moss and for fungi including *Ustilago* and *Fusarium* (**http://hbz.tamu.edu**). For this they are using a proprietary PAGE fingerprinting method and enzyme kit. The soybean physical map has 95 322 fingerprinted BACs covering 11.8X soybean haploid genomes. This map has been integrated with 256 genetic markers on 20 linkage groups and 313 microsatellites will soon be

Copyright © 2002 John Wiley & Sons, Ltd. 

added to this. They are using multiplex hybridisation methods to integrate ESTs in batches of 512 to confirm the map. The map has been used to develop new genetic markers (microsatellites, InDels and SNPs) in regions of the genome that lack conventional genetic markers. They are also making a comparative map of a wild strain, they have found a homologue of a *Glycine max* gene of interest, but it was surrounded by different genes. In their two regions of interest, they have seen 1.5 genes/10 kb and that only 50% of genes were covered by the EST resource, indicating a need for more ESTs.

**Don Langmore (Rubicon Genomics)** presented their **OmniPlex technology and its applications in functional genomics**. This direct genomic sequencing (requiring no cloning) can be used for sequencing transgene junctions, insertion mutagenesis junctions and gene regions (based on EST data) in a range of bacteria and eukaryotes.

**Lynn Jablonski** demonstrated the use of **tools from Integrated Genomics Inc. for comparative analysis and functional reconstruction**. She spoke mainly about ERGO, a database incorporating public and proprietary sequence data, which is designed for use in discovering interactions within the cell. The database expands on the PUMA and WIT databases for metabolic reconstruction.

**Michael Udvardi (Max Planck Institute, Golm, Germany)** presented work using **transcriptome and proteome analysis for the understanding of organ differentiation**. He detailed the current knowledge of plant cell differentiation during nodule formation in leguminous plants, highlighting the role of transcriptomic, proteomic and metabolomic analyses in his group's most recent discoveries (see Trevaskis *et al.*, this issue).

**Bradley Till (FHCRC, USA)** presented TILLING (**Targetting Induced Local Lesions IN Genomes**). This method uses EMS point mutagenesis, followed by self-crossing and then production of a parallel seedbank and DNA resource for PCR for mutation detection. The mutation detection step relies on CEL1, a novel plant endonuclease that preferentially cleaves mismatches in heteroduplexes between wild type and mutant. This is used to digest the products of gene specific PCR amplification of DNA from mutants, using differently labelled primers. The products of the digest are two fragments (whose lengths should add up to the full size of the expected PCR product), fluorescing at different wavelengths. This allows detection of any mutants on a gel, on which eight samples can be pooled per lane. In a trial of the system, they also detected a natural variation in one of the supplied ecotypes and have gone on to show that it can be used to type accessions for SNPs. They have developed PARSESNP, to store information on the mutations, such as which nucleic acid change is involved, and the effect. Backed by NSF funding, the group have embarked on an *Arabidopsis* TILLING program (**http://blocks.fhcrc.org/~steveh/Welcome_to_ATP.html**) in which they can screen ~12 000 samples/day/1 kb region. They have also developed a tool, called CODDLE, which will suggest the best region for TILLING within a gene of interest. TILLING is now being applied to a wide range of organisms.

**D.B. Goodenowe** spoke about the **metabolic profiling approach of Phenomenome Discoveries** Inc. They take a non-targetted approach, detecting just those metabolites in the samples, rather than trying to detect all metabolites. They directly infuse samples into an ion cyclotron mass spectrometer, relying on its ability to give very high mass accuracy to allow them to assume that they can extract correct empirical formulas from the data.

**Peter Gresshoff (University of Queensland, Australia)** spoke about his **nodule related gene discovery and expression profiling** work. His group are interested in root development and the establishment of nodules. There are some genes known to be involved in this process, such as those in the NOD factor signal transduction pathway, however, not all of the genes are known and the regulatory mechanisms are also not completely known. They have taken an insertional promoter trapping approach, using a promoter-less GUS reporter gene. This gives information on gene expression patterns and regulation of genes. They have found an early gene, which has a role in vasculature, this gave mutants with only one nodule. They also identified a lateral root and nodule specific insertion and are currently working out which gene's promoter is causing the pattern. In another approach, his group have made a microarray, with 4000 unique ESTs and other genes represented. Using this they identified 10 differentially expressed genes which they verified using real-time RT-PCR and Northern analysis. In most cases the results matched well, plotting the correlation of the array with qRT-PCR gave a very good correlation score of $r^2 \sim 1$ with linearity over 6 logs.

Copyright © 2002 John Wiley & Sons, Ltd. 

### Databases, gene systematics and nomenclature

**Rolf Apweiler (EBI, UK)** gave the first talk in this workshop. Rolf outlined the **status of proteome resources at the EBI**. He began by describing the SWISS-PROT and TrEMBL protein sequence databases and explained that these resources were now annotated with over 60 000 Gene Ontology (GO) terms. Rolf discussed the InterPro project to link individual proteins to their entries in a range of protein databases. He announced that SMART and TIGRFAMSs had now joined the initiative, with SCOP, CATH and superfamilies to join in 2002. Rolf introduced Clus+Tr, a new project to cluster complete proteomes using a Smith Waterman pairwise comparison. So far, 45 proteomes have been analysed, with the remaining SWISS-PROT+ TrEMBL proteins to be clustered in 2002 (within the Decrypthon project of Telethon). Finally, Rolf looked to the future with a description of the forthcoming Integr8 project. This would provide a comprehensive resource linking EBI databases such as EMBL, SWISS-PROT + TrEMBL, ArrayExpress and IntAct (a protein interaction database) with non-EBI databases. Web access, algorithm development and training would be key to developing a user-friendly system.

The next speaker was **Chris Town (TIGR, USA)**, who discussed the construction and annotation of ***Arabidopsis* gene families at TIGR**, by developing a domain-based approach. He first analysed all domains in the *Arabidopsis* proteome and subsequently clustered proteins into paralogous groups based upon their shared domains. Chris explained that HMM profiles, obtained by matching Pfam HMM profiles against the *Arabidopsis* proteome and then combining the hits with the Pfam seed profile, were matched again to *Arabidopsis*. He found that this approach was more sensitive than using the original Pfam profiles and gave a better seed profile. His results showed that 929 Pfam domains had at least one hit to *Arabidopsis*. These profiles, together with 4,484 novel domain alignments, were used to search *Arabidopsis* and all resulting domain matches and alignments were stored in a relational database. The database contains 4070 families linking 17 684 proteins and leaving only 8515 singletons.

**Jo Dicks (John Innes Centre, UK)** described the current status of the **UK Crop Plant Bioinformatics Network** (UK CropNet). She outlined the crop genome databases for Arabidopsis, Barley, Brassicas, Forage grasses and Potato that are curated and developed as part of the UK CropNet project and explained that the UK CropNet site mirrors many of the US crop genome databases. Jo introduced the new CropSeqDB database that links public sequence on major UK crops to the Arabidopsis database. She discussed access to the data, through the well-established AceBrowser interface. Jo also previewed the new Java/CORBA GFace interface, developed within the UK CropNet project, that will allow fully interactive access to these and other ACEDB databases across the Internet. Finally, Jo described UK CropNet's comparative genome analysis effort, including a comparative mapping database, ComapDB, various Java display tools for comparing genomes and the ARCADE tool to make comparative queries from multiple databases across the Internet.

**Mary Polacco (University of Missouri, USA)** discussed the **MaizeDB database**, focussing on a functional genomics perspective (see also Polacco *et al.* this issue). Mary explained that MaizeDB integrates data from 918 maps with nearly 12 000 loci and many other types of data including genetic stocks, probes, gene products, phenotypes, images and references. The database also includes over half a million links to external databases including ZmDB, TIGR, CUGI, GenBank, SWISS-PROT, GRIN, GrainGenes, RiceGenes and PubMed. She outlined a comparative map utility developed in 2001 to make comparisons based on the same locus, probe or GenBank accession. She also added that this would be enhanced in the future to allow comparisons based on sequence similarity, phenotype and trait. Mary announced several forthcoming improvements to MaizeDB, including sorghum maps, maize QTL maps with trait information and the rice RGP EST map with over 6000 markers.

The final speaker of the workshop was **Cheryl Granger (Iowa State University, USA)**, who described her analysis of **duplication and divergence in the soybean transcriptome**. She explained that she chose to examine soybean genome structure using an EST approach, to discover whether multiple genome duplications had occurred in the evolutionary history of this organism. She based her analysis on random sampling from over 216 000 soybean ESTs from the dbEST database. Cheryl found that while 43% of the sampled ESTs did not match any others, 29% and 13% fell into groups of two and four related ESTs respectively. This provided evidence

Copyright © 2002 John Wiley & Sons, Ltd. 

for multiple gene duplication. Cheryl studied further 144 pairs of related ESTs from the two-groups and found that most sequence changes arose from small gaps or synonymous mutations. She concluded that many duplicated proteins were therefore functionally redundant.

### Controlled vocabularies/ontology for databases

**Michael Ashburner (EBI, UK)** gave an update on the work of the **Gene Ontology (GO) Consortium** (**http://www.geneontology.org/**). The GO is free to industry as well as academia; there are several commercial users, and already some of them have produced add-on tools. The ontology has three organising principles: Molecular Function (the elemental activity or task of a gene product), Biological Process (broader biological goals or objectives in the cell), and Cellular Component (subcellular structures, locations, and macromolecular complexes). All GO terms are unique and are defined with unique identifiers (definitions of the terms are still being written). The terms are organised in a directed acyclic graph (DAG) structure, which allows each child term to have more than one parent term. They use instance (is a, '%') and partative (is part of, '<') relationships between terms. Evidence codes are used to denote the source of inferences made (eg: inferred from mutant phenotype). There are still many compound terms that need to be broken down (disambiguated); this is currently underway. Automated annotation tools are available that can add GO terms; several are produced by companies, including Celera and DoubleTwist. There are also GO browsers, including AmiGO and QuickGO (from the EBI, with links to InterPro etc.). 10 different organism databases are currently using GO terms, and TIGR are reannotating their bacterial genomes with GO. To date, GO is available in MySQL and PostGRES and also Oracle.

**Leszek Vincent (University of Missouri, USA)** presented the **Plant Ontology Consortium**. Their aim is to find a way to describe gene structure, function, phenotypes and parts of plants and cells etc. in a controlled way, allowing searching across the many plant databases that are available now. In the first instance the participants (from Gramene, IRIS, MaizeDB and TAIR) plan to extend the GO terms to cover monocots (see The Plant Ontology Consortium, P).

**Pankaj Jaiswal (Cornell University, USA)** spoke about **Gramene, a comparative grasses database**. Gramene extends the scope of the RiceGenes database, with curated physical and genetic maps, mutant, strain and phenotype data, and comparative maps with other grasses. The team are integrating GO terms into the annotation of 4000 rice genes, and are developing a Trait Ontology as part of the aims of the Plant Ontology Consortium (see Jaiswal *et al.*, this issue).

**Harold Drabkin, (Jackson Laboratory, USA)** spoke about the **Mouse Genome Informatics** resource (MGI), which includes the MGD, GXD, MGS and MTB databases. Within these databases there are many cases of synonyms; they need to standardise these vast datasets and to make sure that all terms have definitions and unique identifiers, whilst allowing for synonyms. MGI has many search forms and provides a GO browser to help users find a GO term to search with. They also have an anatomical dictionary, which is a controlled vocabulary organised by developmental stage and organ (this does allow for synonyms). Their allele and phenotype resource is to switch to using a controlled vocabulary of phenotypic classification terms. In the future, their GO browser will need to have links to genes/items annotated with that term.

**Richard Bruskiewich (IRRI, The Philippines)** presented the **Microarray Gene Expression Database** (MGED) on behalf of their members (**http://www.mged.org/index.html**). MGED is an academic and industrial consortium aiming towards an agreed format for microarray data. Version 1.0 of their minimal information about a microarray experiment (MIAME) was released in July 2001. Version 1.1 was to be discussed at the fourth MGED meeting in February. MIAME makes recommendations on how much information should be supplied about an array experiment, such as on the experimental design, array design, samples and hybridisation conditions. To enable exchange of microarray data between different systems, they have also developed a microarray gene expression mark-up language (MAGE-ML) and data exchange model (MAGE-OM). MGED also has working groups for designing an ontology for describing samples used in microarray experiments, and developing standards for normalisation.

**Jonathan W. Corson-Rikert (Cornell University, USA)** spoke about his work to develop **an integrated controlled vocabulary for the Cornell Genomics Initiative digital library**. A web-portal is

Copyright © 2002 John Wiley & Sons, Ltd. 

planned for the initiative, however the audiences involved, and their goals, are diverse. To make this workable, a common terminology is required, to simplify the task of generating metadata. Individual existing thesauri were not good enough separately, and a process of choosing a start point and adding terms as they were encountered on departmental pages was seen to be slow and flawed. The approach he selected then was to import multiple thesauri: MESH terms, GO terms and the NAL and FAO agricultural indices. This resulted in some inconsistencies and some 'clutter', which required some human intervention. The result is the Cornell Federated Thesaurus (**http://rikert.mannlib.cornell.edu/multith/**). The terms are linked to their source and searches of external resources such as Entrez and SRS with that term have been retained for each descriptor, any broader and narrower descriptors from the source are also retained.

### Bioinformatics

**Gane Ka-Shu Wong (University of Washington, USA)** presented a study of **sequence differences between monocots-dicots and plants-animals**, prompted by their analysis of the recently completed *Oryza sativa* (rice) ssp indica genome. They see a gradient in GC content across rice and maize genes, with high GC at the start of genes, dropping down along each gene. In contrast, dicot genes have consistent GC content across their length. The effect comes mainly from GC3 content, which peaks early in monocot genes, but is consistently low in dicot genes (lower than GC1). This extends to codon usage, which is consistent across dicot genes, but biased in monocots. This could explain why dicot gene prediction programs work poorly for monocot genes. The GC gradient could also have affected their ability to find dicot homologues for shorter rice genes.

Comparing plant genomes with animal genomes, they see that plant transposons are mainly intergenic, whereas in human they are mainly intronic. While plants appear to use gene duplication as their main method for generation of diversity, vertebrate genomes have many alternative transcripts, in addition to showing some gene duplication. There are also differences in 'gene poor' regions; in plants these truly are gene poor, with very few genes, whereas in the human genome these are regions with large genes having sparse exons. The large size of animal genes can make them hard to predict, the average human intron is three times longer than the average *Arabidopsis* intron. Looking at transposons in introns shows that it is mainly transposon presence that is causing the longer introns. Animals have exon recognition proteins, whereas in plants, these have not yet been found, it could be that these allow tolerance of intronic transposons in animal genomes.

**Richard Bruskiewich (IRRI, The Philippines)** spoke briefly about the **International Crop Information System** (ICIS, **http://www.cgiar.org/icis**) and the rice version of the resource (IRIS), which cover genealogy management, gene management and taxonomy. IRIS will be integrated with many monocot databases including those of RGP and TIGR, MaizeDB, UK CropNet and Gramene. He also announced the formation of SPRIG: **specialised plant resources for informatics and genomics**, which can be visited at: **http://bioinformatics.org/sprig**. More detail on the aims of SPRIG, and how to join, can be found in the announcement in this issue.

**Guy Davenport (John Innes Centre, UK)** spoke about **data mining and its application to germplasm analysis**. He gave a background to the field of data mining, which can be thought of as a set of techniques used in a exhaustive approach to automatically uncover information from complex datasets. Data mining can be broken down into high level (prediction and description) and low level tasks (including classification, clustering and visualisation). He described and contrasted several types of data mining algorithms such as decision tree induction, rule induction, neural nets, clustering and heuristic methods. Guy is working on an EU funded project, called GENEMINE (**http://www.gene-mine.org**) which aims to find new methods for management of, and querying data in, genebanks. The group are looking at methods for analysing association between markers and traits, to determine the predictive value of markers for traits, and plan to use heuristic approaches.

**Yuandan Lee (TIGR, USA)** presented TOGA, the **TIGR Orthologous Gene Alignment resource**. This grew from their work on ESTs from a wide range of organisms. TIGR has long been assembling ESTs into consensus sequences (TCs) to generate gene indexes for organisms of interest. Using a purely computational approach, TOGA is produced by assembling TCs from different organisms into orthologous groups.

TIGR currently has 15 animal, 13 plant, 12 protist and 6 fungal gene indices. The orthologous

Copyright © 2002 John Wiley & Sons, Ltd.

groups are assigned using data from pairwise comparisons in each direction between every pair of organisms. Only reciprocal best match pairs are assigned as orthologues. To generate a 'TOG' they require achievement of closure, where two genes that form a reciprocal best match between organisms A and B also share a best match gene in organism C. The TOGA database gives annotation information on each gene and species match patterns and a CLUSTALW alignment for each TOG (**http://www.tigr.org/tdb/toga/toga.shtml**).

**Damian Gessler (NCGR, USA)** presented ISYS, **a system for integrating bioinformatic tools and online resources**. Rather than integrating the data they hold, ISYS addresses the interaction and communication between diverse bioinformatic tools, i.e.: application integration. A detailed description of the system can be found in this issue (see Gessler, this issue).

**Marta Matvienko (Intellectual Force, USA)** presented NODPROTS, an interactive **database for mapping of legume nodulins on the *Arabidopsis* genome**. Using FASTA, the group searched against the *Arabidopsis* predicted ORFs with 760 publicly available legume nodule protein sequences. Using PhyloGrapher, the nodulins (and the resulting set of *Arabidopsis* homologues) have been grouped into 134 clusters. 29 of the clusters have no *Arabidopsis* homologues and hence are excellent candidates for investigating the molecular basis of nodulation, which arose in leguminous genera. GenomePixelizer has been used to draw the *Arabidopsis* homologues on *Arabidopsis* chromosomes, as there were ~1250 genes involved, they have been divided into 7 groups, each subgroup represents homologues of 13–15 nodulin clusters. The *Arabidopsis* genes are linked to their legume homologues in GenBank and to their protein sequences in MIPS. All of the data is presented interactively at: **http://www.intellectualforce.com/nodprots.shtml**.

### International Grass Genome Initiative

**Jeff Bennetzen (Purdue University, USA)** co-organiser and first speaker of this workshop, introduced his work on **comparative genome analysis in the grasses**. Jeff's group had carried out DNA sequencing of comparable regions in six grass species. They found more genome rearrangement in this detailed analysis than they would have expected from the RLFP comparative grass framework and found that some regions were particularly susceptible to genomic rearrangement. Jeff explained that different types of changes occurred at different rates, with changes to coding exons the most conservative and with rates increasing in introns, non-coding parts of genes and intergenic DNA through to the fastest changing entities, transposable elements and SSRs. Jeff ended his presentation by touching on potential mechanisms of evolution, including unequal recombination and illegitimate recombination.

The next speaker was **Ramesh Kantety (Cornell University, USA)**. He is working on **comparative maps between wheat and rice**. A low resolution map was made first, using RFLP analysis of genomic and cDNA probes and grass anchor probes. This indicated that there were large blocks of conservation. To make a higher resolution map he analysed ~65 000 wheat ESTs and 1497 rice BACs and PACs representing ~225 Mb of sequence. His analysis filtered wheat EST matches to the rice sequence that had an E-value of less than $10^{-15}$, with a minimum of 100 bp overlap, more than half of the EST aligned, and greater than 80% sequence identity. At this level, more rearrangement was evident; the map showed small patches of other chromosomes on a background that resembled the coarser RFLP data. On the other copies of the (polyploid) wheat chromosomes, the pattern seems messier. He is also working on collections of barley, maize, sorghum and wheat ESTs and has identified SSR containing ESTs. He has clustered the ESTs to make consensus sequences and identified 1500 cross-species 'super clusters'. These will be used for PCR analysis of SSR variation across the species.

**Roger Wise (Iowa State University, USA)** spoke about his work on local clusters of fungal pathogen defense genes in cereals, in particular those for powdery mildew.

The final speaker of the workshop was **Katrien Devos (John Innes Centre, UK)**, who explained that a **comparison of markers between foxtail millet and rice** showed a high level of collinearity whereas pearl millet exhibited a high degree of genomic rearrangement with these species. Pearl millet was more closely related to maize. In maize, genes were often separated by retrotransposons. She also noted that genetic recombination varies greatly with differing physical-genetic distance ratios.

### Rice

**Takuji Sasaki (NIAS, Japan)** opened this session on the International Rice Genome Sequencing Project

Copyright © 2002 John Wiley & Sons, Ltd. 

(IRGSP), which is working on *Oryza sativa* subspecies japonica. He gave an overview of the progress of the **Japanese Rice Genome Sequencing Project** (RGP). The five chromosomes that they are working on are 1, 2, 6, 7 and 8, which are currently at 84, 83, 81, 80 and 76% coverage respectively. So far, RGP has released ~140 Mb of sequence to DDBJ. The RGP has recently switched to a new strategy, which incorporates the Monsanto data. They are integrating the IRGSP BAC map with the Monsanto map to produce a sequence-ready map; the minimal tiling path is extended using a combination of IRGSP and Monsanto fingerprint data. For those Monsanto clones that were sequenced to 5× draft coverage, RGP are producing shotgun data to increase this to 10× coverage. Their initial aim is to have 10× coverage of all clones, which would result in mainly phase 2 coverage, this will provide a draft much sooner than was originally hoped.

**Rod Wing (Clemson University, USA)** spoke about the work of an American consortium covering the **short arms of rice chromosomes 3 and 10**. Their first aim was to produce a sequence tagged BAC fingerprint framework map for the IRGSP. Currently, they have 461 BAC contigs, 248 of which have been anchored by placing ~700 markers onto the map, this covers ~330 Mb (~80%) of the genome. Adding in the Monsanto data brings this up to 90% coverage. Over 85% of their contigs for chromosome 3, and over 90% of their contigs for chromosome 10, are anchored (they have ~20 Mb of draft sequence for chromosome 10). They are currently working on the gaps, including using FISH to size them.

**Bin Han (National Center for Gene Research, China)** presented the work of the **Chinese rice chromosome 4 project**. Chromosome 4 is highly heterochromatic, ~45% of it appears condensed by FISH. Having already done a lot of work on the subspecies indica, they chose to use a clone-by-clone strategy, based on their physical map, using anchored indica BACs as their starting points. They have built a physical map of the japonica chromosome 4 using data from IRGSP and Monsanto. They have 11 contigs giving 95.1% coverage of the chromosome and the vast majority of their sequence data from these is finished, or in phase II. They have developed an automated annotation package which allows manual modification, and are comparing the indica data with the japonica data.

In addition to playing its part in the sequencing of japonica, **TIGR** hosts a selection of **resources for rice**, **Robin Buell (TIGR, USA)** gave a tour of what is available at **http://www.tigr.org/tdb/e2k1/osa1/**. Their *in silico* mapping of rice BACs has given good coverage of all but the 'orphan' chromosomes, 9 and 11. Their annotation approach makes use of five gene prediction programs (FGENESH appears to perform best for rice), and several databases, to aid gene identification. In addition to their gene index, they have rebuilt their rice repeat database, which includes centromeres and telomeres, transposons, microsatellites, retrotransposons and predictions based on data from maize and related cereals. There are over 30 000 entries comprising ~7.5 Mb of sequence. The BLAST server offers searches of the annotated data, the repeat data and the gene index. They are currently identifying monocot and dicot homologues using genome-wide low stringency alignments with all available plant transcripts. Higher stringency comparisons to find orthologues have resulted in 6654 rice genes being placed in their orthologue resource, TOGA.

The last talk of the sequencing session generated much excitement, as **Jun Yu (Beijing Genomics Institute, China)** announced the imminent publication of **a draft genome of the rice subspecies indica**, completed at the very end of 2001 by a Chinese consortium, The Hybrid Rice Genome Project. The chosen line is the pure indica parent of the vigorous hybrid 'Super-Rice', their aim was 6× coverage plus 1 million ESTs. The other (mixed origin) parent will be sequenced to around 4× coverage (plus 10 000 ESTs) later in the project. If the two datasets together then identify 95% of rice genes, they may not continue and finish the gaps in their drafts. The genome size is over 466 Mb, they have around 361 Mb in contigs, and 78 Mb of fully repeat masked contigs. They estimate that they have 92% coverage and that ~50% of the genome is repeats, including gene families. Simple repeats make up just 1.7% of the genome; these make up ~3% of the human genome. In animal genomes exon dense regions, with clustered small genes, and exon poor regions, with large genes with sparse exons (which have been described as gene deserts) are seen, however, in plant genomes there are gene clusters and true gene deserts, which have no gene content at all. Another difference is that plant repeats are mainly intronic, very few are in intergenic regions. They also saw a major difference in GC content between monocot and dicot genes.

Copyright © 2002 John Wiley & Sons, Ltd. 

Whereas dicot genes tend to have consistent GC content across their length, monocot (rice) genes showed a gradient of GC, with very high GC content at the 5′ end of genes, dropping down across the genes towards their 3′ ends. 80.6% of *Arabidopsis* genes have a rice homologue, and 49.4% of rice genes have an *Arabidopsis* homologue, it is typically the smaller genes (even those with good evidence that they are true genes) that fail to find a homologue. Their current total gene count is 75 659, 53 398 of these have start and stop sites, 3405 have no start or stop defined and the remainder have only the start, or the stop, site located. The distribution of these genes amongst functional classes is similar to *Arabidopsis*, the main differences being more genes for external protective structure in *Arabidopsis* and more genes relating to cell death in rice. The sequence data will be submitted to GenBank and will also be available from **http://btn.genomics.org.cn/rice**.

In the Rice Functional Genomics session, **Yongbiao Xue (Institute of Genetics and Developmental Biology, China)** presented the **Chinese Rice Functional Genomics Program**. The program has several parts including microarray analysis of expression, expression knockdown by antisense and a functional study of transcription factors. More details of the program can be found in the accompanying review (Xue and Xu, this issue) in this issue or by visiting **http://www.rifgp.ac.cn**.

A project to produce **rice deletion mutant populations** was described by **Guo-Liang Wang (Ohio State University)**. Existing resources are mainly insertion lines in japonica, but their approach has been to use chemical and radiation induced mutagenesis, which allows rapid screening for mutants by PCR of pooled samples. In a pilot study they DEB mutagenised an Xa21 resistant plant, producing 500 mutant lines. All of the lines showing complete susceptibility to Xa21 showed deletions in the Xa21R gene. They are now looking at salinity, drought and disease responses and have already isolated some lines with increased resistance to a range of bacterial pathogens. Across all of their experiments so far they see mutations of up to 200 kb using DEB treatment, although some are just point mutations or a few bp long. They predict that there are 10–50 mutations per mutant line as a result of the high doses they are using. They have used arrays to detect the larger deletions and suggest the TILLING technique would be suitable for detecting the point mutations. They have produced a database of their mutants (made with DEB, EMS and other mutagens) which holds data on over 30 000 lines.

**Ashikari Motoyuki (Nagoya University, Japan)** described work on the isolation and characterisation of the **rice green revolution gene**, ***semi-dwarf 1***. In the 1960s, the International Rice Research Institute produced a shorter, high producing hybrid rice that became known as ‘miracle rice’, averting the predicted world food shortage in the 1960s–70s. This *sd1* line produces more grains in response to nitrogen in fertiliser, without the usual concomitant elongation of the culm, which leads to lodging (falling over in windy and rainy conditions). One of the most obvious potential reasons for dwarfing would be a mutation relating to the Gibberellins (plant growth hormones). Adding GA1 did complement *sd1*, but mutations in all six of the known GA synthesis genes were shown to cause a severe dwarf phenotype. The *sd1* line was shown to have reduced levels of GAC20 oxidase compared to wild type, which indicated a mutation in a redundant C20 oxidase. The team found a novel gene with homology to the known C20 oxidase gene, which mapped to the same locus on chromosome 1 as *sd1*. They have found differences in this gene compared to wild-type, in five sd1 lines and shown that complementation with the wild-type locus produces a wild-type phenotype. The gene does show a different expression pattern than GAC20ox1, indicating that it is not wholly redundant.

### Arabidopsis

This session was opened by the Chair, **Renate Schmidt**. She explained that since the genome of *Arabidopsis* was completed at the end of 2000, the study of natural variation in the genome, and functional analyses (such as metabolic profiling and mutagenesis), seemed ideal choices for the theme of the session, demonstrating the wealth of information to be had from the genome and downstream analyses.

A study into **natural variation in light responses and flowering** in *Arabidopsis* was described by **Julin Maloof (Salk Institute, USA)**. He argued that the study of natural variation can allow the discovery of genes which are redundant, or have multiple functions, or make only a small contribution to a trait, leading to the dissection of complex traits.

Copyright © 2002 John Wiley & Sons, Ltd. 

Plants have a complex set of photoreceptors and exhibit traits such as phototropism (light-induced directional growth) and avoidance of shade (in plants in direct light). Germinating plant shoots grow under the soil as etiolated hypocotyls, and only open their cotyledons after detecting the soil surface. Comparing strains from across the world showed that plants from near the equator were less sensitive to light (being adapted to higher light intensity) than plants from nearer the poles. Testing these plants with different light conditions (white, blue, red, far red and dark) showed that this response was not light specific, but wavelength specific. One far red sensitive line was shown to have a point change in the chromophore region of the PHYA photoreceptor gene. Their work on variation in flowering time has led to the mapping of a major QTL for flowering in short days. This QTL, *FLOWERING1*, appears to be the result of a deletion of a MADS-box gene.

**Jose Martinez-Zapater (UAM, Spain)** presented an analysis of **natural variation in freezing tolerance** in *Arabidopsis*. Plants can exhibit a cold hardening response; on exposure to cold, but not freezing, temperatures, the plants acquire an ability to survive freezing temperatures that would otherwise kill them. In *Arabidopsis*, the $LT_{50}$ is $-5°C$, and in acclimatised plants, $-8°C$ (a very modest response compared to trees, which can survive $-30$ to $-40°C$). Acclimation in addition to short days increases survival to $-10°C$, so there must be two mechanisms at work. Over 80 genes had already been shown to be cold regulated, but they wanted to know which of these were responsible for the natural variation seen in the response. Looking at accessions from around the world they found one from Cape Verde, near the equator, which had almost lost the acclimatisation response. They crossed this with Landsberg erecta and tested the tolerance of the progeny. Their genetic analyses pinpointed three QTLs on chromosomes 1, 4 and 5. The locus on chromosome 4 was shown to be due to polymorphisms in two CBF genes between these two strains.

**Daniel Kliebenstein (University of California, Davis, USA)** reported on a study of **natural variation in resistance to insect herbivory**. Insect herbivory has a heritability of only 20–30%, with the environment of the plant playing a large role. The group had studied over 10 000 EMS mutants, finding very little variation, when they turned to natural variation, finding far more variation in just 20 accessions. Landsberg erecta was very susceptible, and Columbia was much less susceptible to the predator the team was testing with. Taking a candidate gene approach, they looked at glucosinolate hydrolysis genes. These enzymes only come into contact with their substrates on damage to the plant and degrade them to different end products in different plants. The products act as feeding deterrents for most organisms. This implies that these enzymes must work with another compound, a specifier, which decides which end product is made. They have so far used natural variation studies to clone five of the six genes that control glucosinolate type in *Arabidopsis* leaves, including the one behind the difference between the resistance of Landsberg erecta and Columbia.

**Oliver Fiehn (MPI-MPP, Germany)** presented **metabolomic analyses of *Arabidopsis thaliana* crosses**. The Col and C24 accessions were crossed with a range of accessions, including Ler, Hi and Rsch. Metabolites from leaf discs at the same developmental stages were extracted and derivatised using milder conditions than for their earlier experiments. LC/LC coupled to tandem mass spectrometers (Time of Flight and ion trap) was used for faster metabolite detection. They found $\sim 550$ polar metabolites and $\sim 500$ lipophilic metabolites, but many of the peaks observed were unknown, which requires *de novo* identification. Principle component analysis was used to derive metabolic phenotypes, which were then used to determine relative metabolic distances between accessions. This system separates Ler and C24 well and separates some of the progeny. Looking at shifts in pathways they see good numbers of significant differences, which can be broken down into trends, such as amines being predominantly down regulated in C24. Looking at ratios of metabolites, they saw that the ile/leu ratio is the same in both parents and progeny, whereas sugar patterns tend to be more variable. Calculating metabolite-metabolite correlations and plotting a graph of these showed correlation between metabolic pathways or cycles and their positions in the graph. Comparing the graphs of different accessions shows differences in the regulation of metabolic pathways.

**Xin Li (University of British Columbia, Canada)** presented **Delete-a-gene, a reverse genetic method** using fast neutron bombardment to generate mutant plants, which can be rapidly screened by PCR to identify lines with a mutation in a gene of choice (See Li and Zhang, this issue).

Copyright © 2002 John Wiley & Sons, Ltd. 

### Microbial functional genomics

**The Chairman, Peter Johnson**, started the session with the news that over $10 M will be made available for microbe genome sequencing projects in 2002. He also announced that next year, the microbe sessions will split from PAG and join the ASM/TIGR conference on microbial genomes.

**Carie Harwood (University of Iowa, USA)** spoke about ***Rhodopseudomonas palustris***, a versatile phototrophic bacterium. The genome of this purple, non-sulphur α-proteobacterium was sequenced at the DOE Joint Genome Institute. *R. palustris* converts light into chemical energy, and fixes $CO_2$ and Nitrogen. It also degrades and recycles lignins. It is one of the most metabolically versatile bacteria so far described, and can switch between four main modes of metabolism depending on the conditions. The genes involved in sensing when to switch, and those that regulate these and other pathways (such as stress responses) are of great interest.

**Nicole Perna (University of Wisconsin, USA)** presented her work on **comparative genomics of enterobacterial pathogens**. There are currently 4 *E. coli* strains with complete genome sequences and another 3 are underway (including *S. flexneri*). There are also 3 *Salmonella* and 2 *Yersinia* genomes, and 2 *Erwinia* genomes are underway. Comparing *E. coli* 0157 against K12 they see a 4.1 Mb conserved backbone (98.5% identical), 0157 has 1.34 Mb of additional, 0157 specific sequence and there is 0.53 Mb of sequence specific to K12, in each case, the specific sequence is distributed all over the genome as islands. 0157 has more phage like regions; the locations of insertions are not conserved and different families of insertion sequences are seen. Genes specific to 0157 include fimbrial adhesins, non-fimbrial adhesins, iron transport genes; there are several pathogenicity islands, with some less than 2 kb in size. The islands have atypical composition, with broader GC content, indicating lots of horizontal transfer has occurred. They use PCR analysis of patches of the genome to fingerprint strains. They do see 0157 and K12 groupings, but there is also variation within these.

**Alan Collmer (Cornell University, USA)** spoke about **genomics and phytopathogenicity of *Pseudomonas syringae* pv. Tomato DC3000**. This bacterium causes leaf spots in tomato and *Arabidopsis*; there are over 40 pathovars, some of which affect beans and tobacco. *P. syringae* is a Gram negative stealth pathogen (it can be detected in apparently healthy plants). The sequence of this strain is almost complete and there are plans to sequence one of the bean specific pathovars. Their aim is to identify novel avirulence genes (effectors of Hrp). Known genes of this class show no common motifs, but always have an upstream Hrp box. This sequence is too degenerate for sequence searches, so they have designed an assay to detect functional Hrp boxes. This approach has located four new candidate effectors. This expanded the number of known functional Hrp boxes to 18, which they used for hidden Markov modelling to search across the genome for more. This has allowed them to find several high scoring boxes, all of which have been shown to be in front of genes showing expression in a microarray assay. Using these new proteins and the known ones they have constructed 5 rules for detecting the N-termini of avirulence genes, these have predicted 15 novel candidate effectors, and have been shown to work for subsets of *Xanthomonas* and *Yersinia* effectors.

**Bruce Seal (USDA/ARS)** discussed the genomic sequence analysis of avian paramyxoviruses and their phylogenetic relationships with other members of the family *Paramyxoviridae*.

**Jim Liao (UCLA, USA)** presented work on **DNA microarray analysis and metabolic engineering in *E.coli***. He took the 52 publicly available *E.coli* array datasets and added in 14 datasets from his own lab, covering 9 different conditions in total. He then looked at potential operon pairs, with a view to predicting operons. He took training sets of known operon genes and known non-operon genes, and made a contour plot of pairwise correlation and distance between genes, which highlighted a non-operon zone and an operon zone. He found that expression data is the best predictor of an operon, whereas distance is better for predicting non-operon. In each case, adding in the other data makes no real improvement. Part of the problem arises from the types of array experiments being done, often they test specific conditions which would stimulate only a small subset of operons. Another issue is noise, looking at known operon pairs, some were very noisy across the datasets. Including an allowance for this has improved their predictions, as does their continual addition of more datasets. Predicting regulons is more difficult. Looking at growth on glucose compared to acetate, which relates to the conversion of pyruvate to phosphoenolpyruvate (PEP), they saw strong upregulation of an apparently unrelated gene (PPS). This gene was shown to have

Copyright © 2002 John Wiley & Sons, Ltd. 

no phenotype in steady state, but only in the transition phase. Comparing the mutant with wild-type has identified several more genes which appear to be related to the need for PEP.

**Turlough Finan (McMaster University, Canada)** spoke about the **Alfalfa symbiont *Sinorhizobium meliloti***, which is found in Nitrogen fixing root nodules. This bacterium is similar to *Agrobacterium tumifaciens*. An international consortium has recently finished sequencing the genome which consists of a chromosome and two plasmids. It has been shown that one of these plasmids can be almost completely deleted to no apparent ill effect, however deletion of one region does result in small chlorotic plants with nodules unpopulated by bacteria. This region has the Nitrogen fixation genes and phosphate uptake and utilisation genes. The group have designed a cloning strategy using homologous recombination at oriT sequences, they suggest that this might occur naturally and that some insertions might be flanked by these oriT sequences.

**Vivek Kapur (University of Minnesota, USA)** spoke about his work on ***Pasturella multocida***, a pathogen related to *Haemophilus* and *Actinobacillus*. From 266 isolates collected they have identified 72 clones, eight of which are common. The genome project showed that $\sim$40% of genes were of unknown function, and that there were only a few, well ordered repeats. Several putative virulence factors were identified, such as a filamentous haemagglutinin, which is expressed *in vitro* in pathogenic strains but not in non-pathogenic strains. There are also iron acquisition systems, which have prompted them to use arrays to compare WT with treatment with an iron chelator. They are now working on the genes that showed strong induction or repression in these experiments, some of these have transport functions, some are related to energy production and many are unknowns.

**Julian Parkhill (Wellcome Trust Sanger Institute, UK)** spoke about **comparative genomics of bacterial pathogens**. He started by discussing *Salmonella enterica* serovars. The *S. typhi* chromosome shows high colinearity with that of *E. coli*, although there has been some recombination between the RNA genes around ori, and there are several insertions (pathogenicity islands). There are also several complex rearrangements (translocations, inversions and small deletions) and what appear to be the remnants of deleted genes. Comparing *S. typhi* and *typhimurium* highlights many large and small insertions in *typhi*. Looking at the overall picture, there are as many genes moving in these small insertions and deletions as in the big pathogenicity islands. There are also several genes present in both serovars, but rendered inactive in *typhi* by point mutations. This inactivation of genes is also seen in many of those gained in insertions. He suggests that *typhi* has gone through an evolutionary bottleneck in changing from a multi-host gastrointestinal pathogen to a single host, systemic bacterium. *Yersinia pestis* has also swapped from being gastrointestinal to systemic. In this transition it has lost genes for the O antigen and genes required for the flagellum. Three large regions of its chromosome show reversed GC skew, all are flanked by IS elements. PCR amplification across the boundaries confirmed these results, but also indicated that rearrangement at these boundaries happens frequently in isolates. When they compare the genome against *E. coli*, they see more scrambling of conservation than would be expected for the evolutionary distance between them. At the Sanger Institute, they use ACT (the Artemis Comparative Tool) to visualise the comparisons (**http://www.sanger.ac.uk/Software/ACT/**), making detection of insertions, deletions and rearrangements much simpler.

---

The Meeting Highlights of *Comparative and Functional Genomics* aim to present a commentary on the topical issues in genomics studies presented at a conference. The Meeting Highlights are invited and each represents a personal critical analysis of the current reports, which aims at providing implications for future genomics studies.

Copyright © 2002 John Wiley & Sons, Ltd. 

